# Supplementary figures and images for: Rapid detection of Enterococcus and vancomycin resistance using recombinase polymerase amplification
Source: PeerJ. 2021 Dec 7;9:e12561. doi: 10.7717/peerj.12561 (PMC8663621; doi:10.7717/peerj.12561)

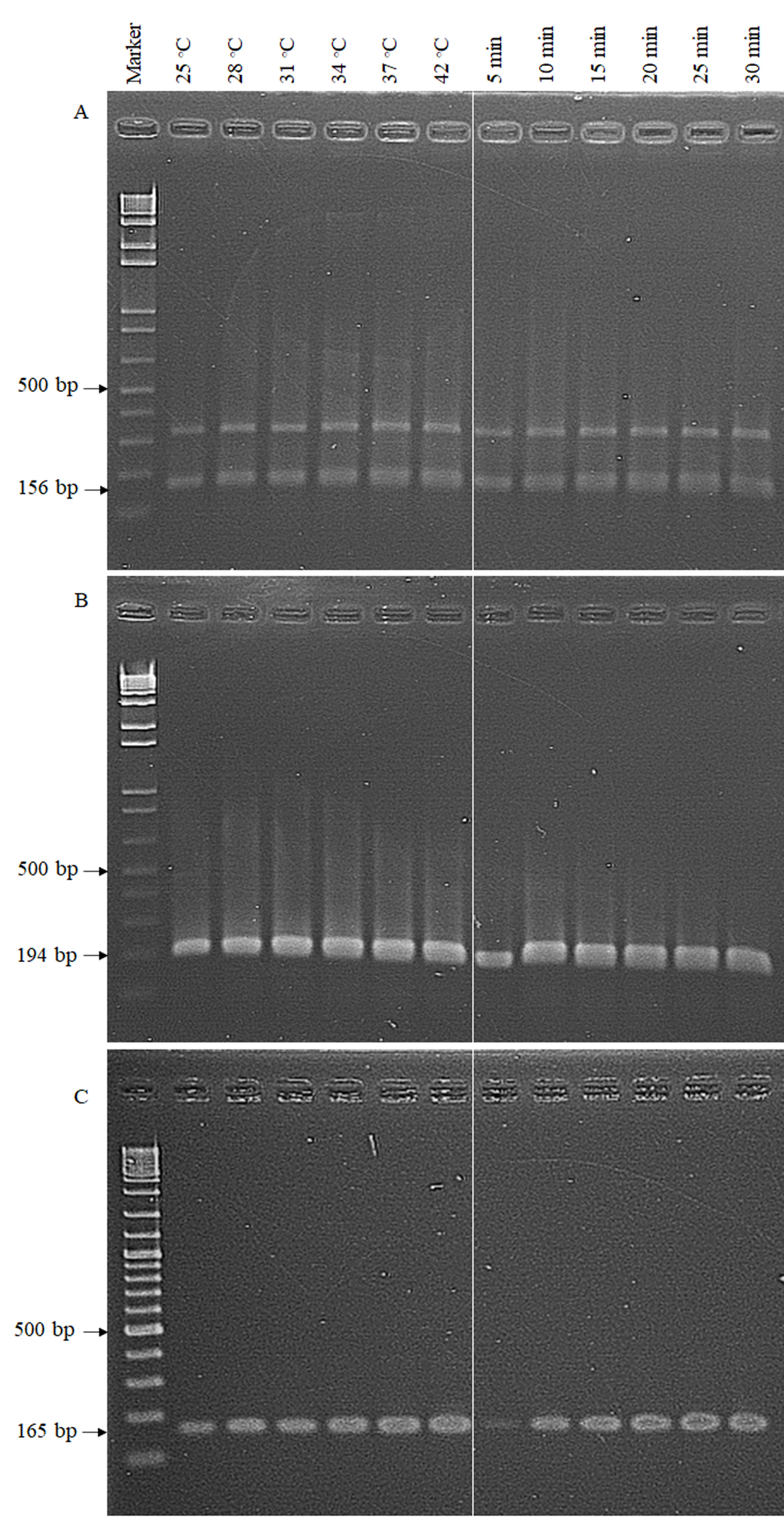

Supplement: Supplemental Information 1 — The RPA-AGE worked well across a broad range of temperatures. An obvious test band could be seen when the reaction time was 20 min at 37° C. Marker, GeneRuler DNA Ladder Mix (Thermo Scientific, California, USA) [file peerj-09-12561-s001.jpg]

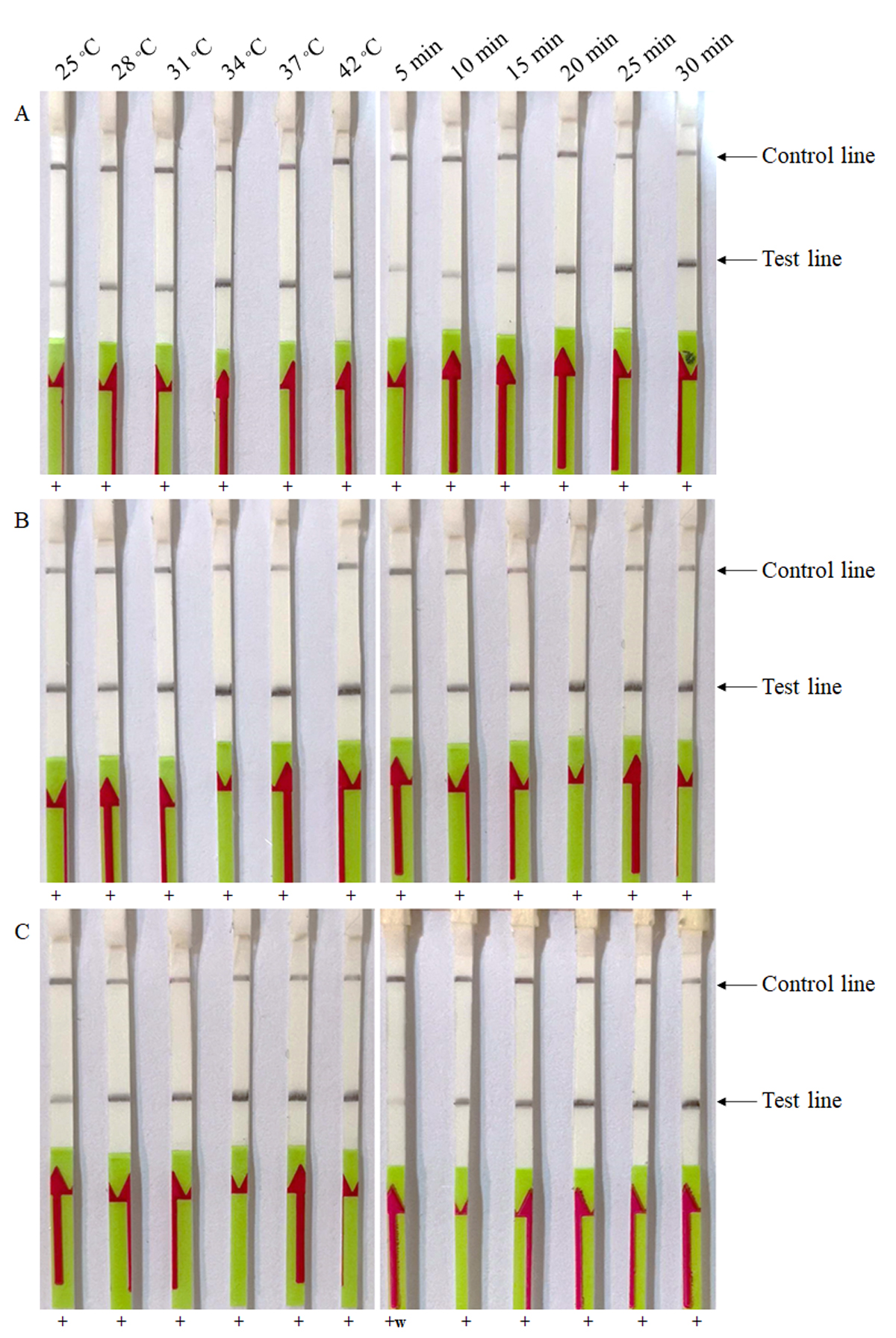

Supplement: Supplemental Information 2 — +, positive result; +w, weakly positive result [file peerj-09-12561-s002.jpg]

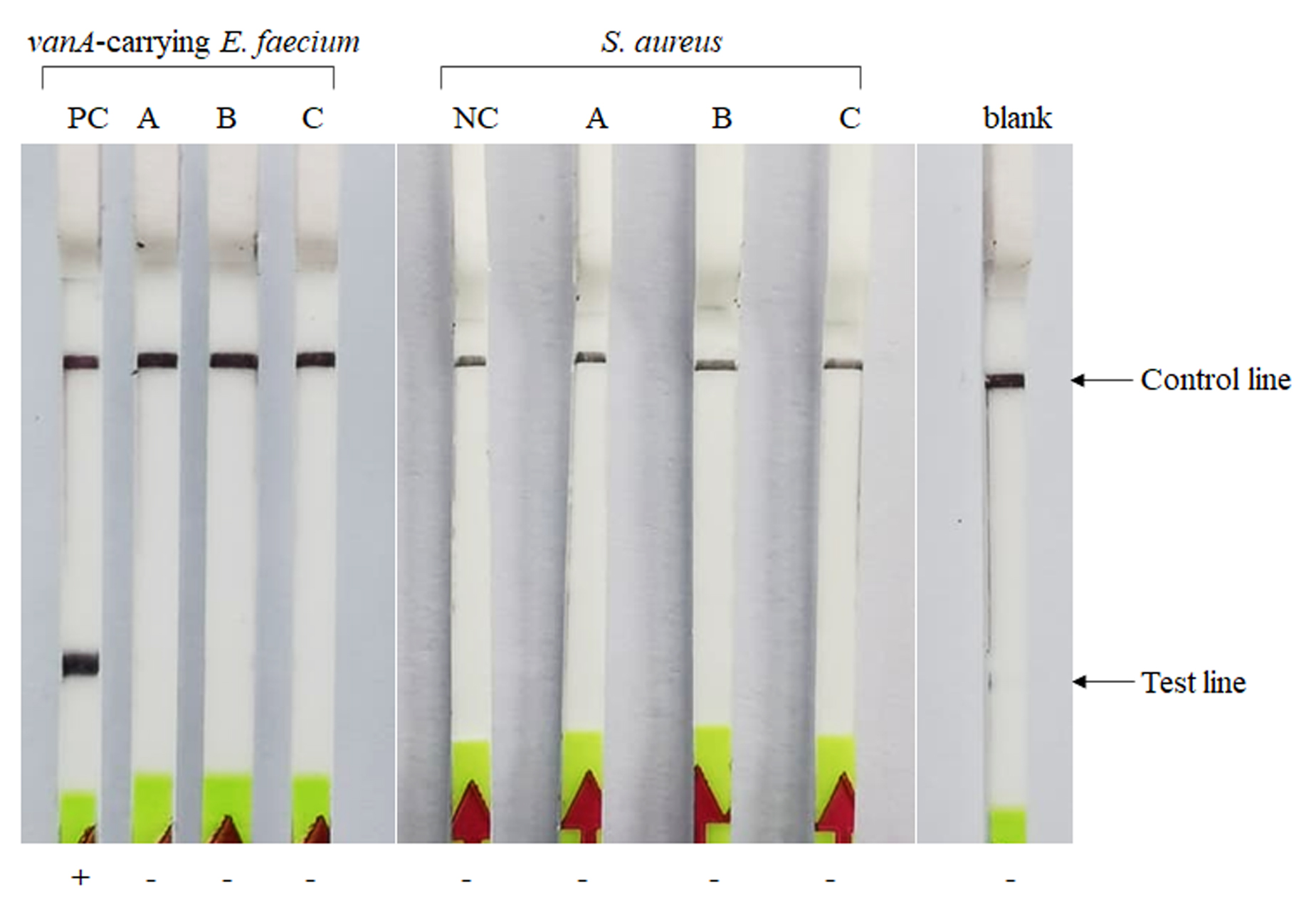

Supplement: Supplemental Information 3 — A, bacteria in stool and bile esculin broth (BE broth); B, bacterial sample in BE broth; C, bacterial sample with stool in sterile water; PC, positive control (DNA of vanA-carrying E. faecium); NC, negative control (DNA of S. aureus); +, positive result; -, negative result [file peerj-09-12561-s003.jpg]
