# Supplementary material for: Rapid detection of Enterococcus and vancomycin resistance using recombinase polymerase amplification
Source: PeerJ. 2021 Dec 7;9:e12561. doi: 10.7717/peerj.12561 (PMC8663621; doi:10.7717/peerj.12561)
Supplement: Supplemental Information 4 — a 95% CI; 95% confidence interval b The number of positive vanB gene was 2 isolates, while negative vanB gene was 72 isolates. [file peerj-09-12561-s004.docx]

**Table S1** Performance of the RPA-LF method for detection of *E. faecium,* and of *vanA,* and *vanB* genes in positive blood-culture and stool or rectal-swab samples using conventional PCR as a reference method

| **Performance characteristics** | ***E. faecium*** | | ***vanA*** | | ***vanB*** |
| --- | --- | --- | --- | --- | --- |
|  | Blood culture | Stool or rectal swab | Blood culture | Stool or rectal swab | Blood culture |
| Sensitivity  (95% CI)^a^ | 100  (92.5-100) | 100  (92.8-100) | 100  (87.6-100) | 100  (87.9-100) | 100  (19.8-100^b^) |
| Specificity  (95% CI)^a^ | 100  (94.4-100) | 100  (93.7-100) | 100  (95.6-100) | 100  (95.4-100) | 100  (93.7-100) |

^a^ 95% CI; 95% confidence interval

^b^ The number of positive *vanB* gene was 2 isolates, while negative *vanB* gene was 72 isolates.
